# Using the Electrocardiogram for Pain Classification under Emotional Contexts

**DOI:** 10.3390/s23031443

**Published:** 2023-01-28

**Authors:** Pedro Silva, Raquel Sebastião

**Affiliations:** 1 DFis, University of Aveiro, 3810-193 Aveiro, Portugal; 2 IEETA, DETI, LASI, University of Aveiro, 3810-193 Aveiro, Portugal

**Keywords:** electrocardiogram, emotional contexts, machine learning, pain, physiological features

## Abstract

The adequate characterization of pain is critical in diagnosis and therapy selection, and currently is subjectively assessed by patient communication and self-evaluation. Thus, pain recognition and assessment have been a target of study in past years due to the importance of objective measurement. The goal of this work is the analysis of the electrocardiogram (ECG) under emotional contexts and reasoning on the physiological classification of pain under neutral and fear conditions. Using data from both contexts for pain classification, a balanced accuracy of up to 97.4% was obtained. Using an emotionally independent approach and using data from one emotional context to learn pain and data from the other to evaluate the models, a balanced accuracy of up to 97.7% was reached. These similar results seem to support that the physiological response to pain was maintained despite the different emotional contexts. Attempting a participant-independent approach for pain classification and using a leave-one-out cross-validation strategy, data from the fear context were used to train pain classification models, and data from the neutral context were used to evaluate the performance, achieving a balanced accuracy of up to 94.9%. Moreover, across the different learning strategies, Random Forest outperformed the remaining models. These results show the feasibility of identifying pain through physiological characteristics of the ECG response despite the presence of autonomic nervous system perturbations.

## 1. Introduction

Everyone in today’s world has experienced suffering at some point in their lives. Since pain is a varied experience for each individual due to many various factors, it is difficult for health practitioners to grasp the pain felt by the patient [1].

Pain is a vital nervous system signal that suggests something is wrong within or outside the human body and is an aggravating and confusing sensation that disturbs and makes people uncomfortable. Pain is impacted by many various elements, such as age and gender, and it manifests itself in a variety of forms and intensities, such as burns, ashes, or pricks in a specific place of the human body [1]. When we are in pain, we undergo considerable autonomic alterations, which leads to increased sympathetic outflow. Similarly, pain alters the autonomic regulation of the heart, which may be assessed with a Heart Rate Variability (HRV) analysis to determine the sympathetic and parasympathetic tone [2].

Currently, there is no easy or automatic way to objectively measure pain. Thus, pain evaluation necessitates reliance on patients’ quantitative and/or qualitative descriptions of painful feelings and relies on self-reporting through scales, namely the Visual Analog Scale (VAS) or the Numeric Rating Scale (NRS). The main idea behind these scales is to ask the patient to choose the pain level from 0 to 10, where 0 represents no pain and 10 represents the worst pain imaginable. The issue with these measurements is their reliance on patient knowledge, communication, and pain experience. As a result, it will not operate in circumstances such as unconscious adults or newborns, among others. Therefore, more accurate methods of assessing pain intensity, such as automated pain intensity recognition, must be developed.

Furthermore, emotions can alter the severity of pain perceived, either decreasing or increasing it. For example, if the patient is joyful, the severity of the pain is reduced; if the patient is unhappy, the intensity is enhanced [3]. Thus, when investigating pain, it is advised to consider the patient’s emotional condition.

There are several hurdles in the development of automatic pain identification systems, including (1) the availability of data, which are difficult to obtain; (2) interindividual variances, which frequently account for more variation than the signal of interest, and in a clinical setting, a baseline-only calibration appears to be the most realistic, though group-specific models may be an option; (3) measuring low-intensity pain, which only yields low amplitude responses; (4) dealing with interfering factors and artifacts, such as motion during data collection; and (5) the systems must assess pain and accompanying emotional states concurrently, due to the influence of the latter on pain.

Although several studies have focused on the human body’s response to pain in recent years, namely undertaking the identification of pain based on physiological characteristics, few investigations have addressed the impact of emotions and their involvement in the sense of pain.

As a result, there is a critical need to advance quantification techniques that should be simple, direct, cost effective, relevant regardless of the circumstance, and takes into account the emotions felt during measurement.

This work is organized as follows: Section 2 presents the background on pain induction and related works on the assessment of pain using physiological signals. Section 3 describes the implemented protocol for data collection, along with the materials and methods used in this study. In addition, it also presents data preprocessing and processing techniques, a description of the extracted features, and the machine learning methods employed. Section 4 and Section 4.2.4 present the obtained results and a discussion of these, respectively. Finally, the key findings and future work are exposed in Section 6.

### Challenges and Goals

This work intends to go further in pain classification, aiming at analyzing the physiological response to pain under different emotional contexts and the stability of the physiological response when pain is induced at different time periods. To attain these goals, a pain-inducing protocol with emotion elicitation was implemented at different time periods for the same participants, and several training and testing approaches were proposed.

The first challenge was the construction of a dataset with physiological signals collected during pain induction and under emotional contexts at different time periods, allowing the reasoning on the elements that have the most influence on pain recognition, and contributing to the development of a system that can recognize pain taking the time and the emotional correlates into account.

In addition to the construction of the database, this study aims to analyze the electrocardiogram (ECG) signal during pain induction under different emotional contexts. The main hypothesis is that although different elicited emotions may alter the pain perception of the participants, the physiological response to pain should remain the same. Moreover, most of the existing studies attain the recognition of pain through a multimodal perspective, considering the combination of characteristics extracted from different physiological signals. Thus, grounded only on the most widely used physiological signal, the second challenge is how to derive pain recognition models by relying solely on information conveyed by the ECG and taking into consideration the effect of time span and emotional conditions. This was attained by proposing a session-independent approach to predict pain, which allows us to reason about the stability of the physiological response to recognizing pain.

Finally, the last challenge derives from the fact that the literature provides few pain recognition models using a participant-independent approach. For these, the performance of models is evaluated in data from participants that were not present in the training process. In this work, this goal is accomplished by advancing pain recognition models trained in a leave-one-out procedure with data from both sessions.

## 2. Background and Related Works

To generate and evaluate pain in a controlled environment, three essential parameters are required: a noxious stimulus, a protocol or technique of stimulus delivery, and a standardized way of measuring the response to noxious stimulation [4].

The Cold Pressor Task (CPT) is an example of a method of thermal stimulation, whereby the participant places their hand and forearm in cold water for as long as they can handle, and a slow and gradual pain is induced that dispels quickly after the removal of the limb from the tank [5]. This procedure usually employs water with a temperature between 5 °C and 15 °C, and the lower the temperature the more pain is induced. The use of 10 °C water is very common with children and 6 °C with adults [5,6]. This technique has various advantages, including the absence of elements that might induce weariness, nausea, and unpleasant medical procedures in clinical practice. Furthermore, this procedure is safe, interesting, and efficient, and it allows for control over the position, duration, and strength of the produced stimulation. Additionally, it takes little training to utilize, owing to the nonthreatening character of cold-induced discomfort. However, it has certain drawbacks, such as high methodological heterogeneity in task utilization and pain outcome measurement between research teams and studies [6].

### 2.1. Related Work

Considering the alterations that pain incites in autonomic reactivity, the ECG is the most explored signal in the literature. The ECG is a sort of graph that depicts the electrical activity of the heart, offering a time–voltage chart of the heartbeat. As a result, the ECG is separated into sections based on the electrical activity of the heart, such as the P wave, QRS complex, and T wave [7].

Concerning the physiological analysis of pain, several works in the literature make use of the BioVid Heat Pain Database [8], a multimodal database in which pain was induced at different degrees, as well as emotion elicitation using the dimensional and discrete paradigm. The study’s major goal was to identify the elements and feature patterns that lead to the highest identification rate for pain perception, quantification, and emotional dissociation [8]. There are 90 participants in this database, ranging in age from eighteen to thirty-five years old, thirty-six to fifty years old, and fifty-one to sixty-five years old, with half being men and the other half being women, and each age group had 30 participants. The Skin Conductance Level (SCL), ECG, electromyogram (EMG), and electroencephalography (EEG) were the biosignals acquired using a Nexus-32 amplifier. The pain was induced with a thermode inserted in the right arm, and the participants were seated in a chair with their arms resting on a desk in front of them during the protocol. It is conceivable to evoke quantifiable pain under high control conditions with this technology without producing skin burns. The pain was stimulated for around 25 min, with four different individual pain levels being randomly selected and stimulated 20 times each. Participants expressed happiness, sorrow, rage, fear, disgust, and pain during the procedure. Images and movies were utilized to induce these feelings.

The BioVid Database was utilized in [9], and features were extracted from videos and biosignals. The features were then used to train a Random Forest (RF) classifier. Using just the biosignals, the accuracy in classifying between the baseline and each pain level ranged between 49.1% and 75.6%, with the greatest pain level having the best accuracy and the lowest pain level having the lowest accuracy. These findings are not surprising given that the majority of the participants did not show responses to the low pain stimuli at all. Higher levels of pain result in greater behavioral and physiological responses, which are more easily detected, while the lowest pain stimulus was almost undetectable in their system. In the lowest level of pain, the responses are either too slight to detect from noise, or they are absent entirely. Moreover, except for the lowest intensity level, person-specific models outperformed generic ones in general.

Another study that used this database is [10], retrieving ECG and EMG features, obtained from amplitude (related to the amplitude of the signal waves), frequency (related to the number of occurrences of repeating events per unit of time), stationary (reflecting some form of stable equilibrium), entropy (measuring the disorder of the system), linearity (measuring the proportionality of changes in parameters), and variability (describing the dispersion) before being fed into support vector machines (SVM). However, in this study, pain levels were compared rather than all levels to the baseline. The accuracy varied depending on the type of feature selection used; however, with forward selection, the accuracy was greater than 88%. They also determined that the properties in the categories of linearity, stationarity, variability, and frequency may only be considered good when they are deemed to have more relevance in terms of the length or form of pain. Furthermore, the EMG characteristics appeared to contribute significantly to pain measurement.

Additionally using the BioVida Dataset, the work of [2] advanced a regression algorithm based on recurrent neural networks for the continuous estimation of pain intensity, using Skin Conductance (SC) and HRV measures. Using undersampling to counteract the original imbalanced data, after balancing the different classes of pain levels, the authors reached a MAE of 1.05.

In [11], the authors compared the capacity of a combination of numerous autonomic measures and each parameter alone to discriminate between four levels of pain severity. In this study, 55 healthy participants, comprising 21 women and 34 men ranging in age from 20 to 37 years old, were required to remain supine and avoid any movements for this purpose. During the experiment, four heat intensity levels were randomly delivered for one minute each, with 10 to 15 min intervals between the stimuli. In addition to the pain-inducing techniques, respondents verbally assessed the felt pain intensity every 10 s during the stimulation time, and physiological data such as ECG, photoplethysmography (PPG), and SCL were gathered. The findings of this study showed that all five examined indicators correctly distinguished between no pain and all other pain categories. However, none of the criteria distinguished between the three pain categories. The linear combination of factors, on the other hand, distinguished not only between pain and no pain, but also between all pain categories. Furthermore, while individual autonomic markers do correspond to some extent with various pain intensities, their linear combination differentiates across pain categories better than each parameter alone.

Another work [12] utilized physiologic signals, such as SCL, Blood Volume Pulse (BVP), and ECG, and focused on feature processing. Then, these were placed in a genetic algorithm (GA) to reduce the number of features used by detecting the redundant ones. Posteriorly, the features were transformed into linearly uncorrelated space via Principal Component Analysis (PCA). To classify the data, SVM, linear discriminant analysis (LDA), and k-nearest neighbor (kNN) algorithms were utilized and were evaluated for different datasets, single-signal datasets, and multisignal datasets, as well as for multisubject datasets and multiday datasets.

The authors of [1] proposed data augmentation before the pain classification stage. For this purpose, the authors generated data using Least Square Generative Adversarial Networks. With multibiosignals (EMG, SCL, ECG) for different levels of pain from 85 healthy participants, and by extracting 159 physiological features, the authors also performed feature selection prior to training an SVM-rbf model (SVM with Radial Basis Function kernel), achieving an accuracy of 82.8% for the multiclassification of pain levels.

Going beyond the pain recognition, the work of [13] proposes a framework for feature extraction methods allowing for a fair comparison of the performances of feature extraction and feature learning approaches. The authors concluded that simple feature engineering approaches, relying on features extracted from the signals based on expert knowledge, lead to better performances than deep learning approaches, and that more complex deep learning architectures do not necessarily outperform simpler ones. Moreover, according to the survey used in [14], the works that rely on neural network architectures for pain recognition used features extracted from video or audio content. On the other hand, when attaining pain recognition through physiological modalities, several authors rely on machine learning algorithms such as SVM, LDA, kNN, regression, Decision Trees (DT), and tree ensembles.

## 3. Materials and Methods

This chapter details the protocol’s related aspects, as well as the methods used for data analysis.

### 3.1. Experimental Protocol for Data Collection

This research was undertaken to measure the effects of emotional contexts, where individuals were subjected to pain while different emotions were elicited, to determine the differences in the physiological responses to pain. Thus, a specific experimental protocol was designed with the goal of discovering which changes pain causes in human bodies and how emotional contexts affect these.

It is important to point out that this procedure was performed during the SARS-CoV-2 pandemic, which meant that adjustments had to be made to diminish the risk of infection for the participants and researchers conducting the study, such as the use of protective equipment and disinfection of the space and hands.

#### 3.1.1. Inclusion and Exclusion Criteria

Volunteer participants were sought from the university students’ community according to the following criteria: (1) age superior to 18 years and inferior to 50 years; (2) not displaying severe mental illness or neurological diseases; (3) not suffering from any ailment which causes chronic pain; (4) ability to interpret and answer the self-report measures; and (5) willing to offer informed consent and participate in this study.

Due to the CPT procedure, participants having a history of cardiovascular illness and Raynaud’s disease, seizures, frostbite, cuts, sores, or fractures on the hand and/or on the forearm to be immersed were disqualified. After the selection, all the participants were submitted to psychological evaluation accompanied by numerous questionnaires (adapted and validated to the Portuguese language) assessing personality, stress, anxiety, and health.

#### 3.1.2. Ethical Considerations

The CPT is recommended for this purpose and is considered ethical to use as it is a valid way to attain the established goals, does not cause collateral damage such as tissue damage from psychological trauma, and the stimulation is considered very low. To determine if this procedure is ethical or not, one must carefully analyze the benefits and the risks. Even though the CPT causes pain, the participant is completely in control over the procedure, since the participant can withdraw their forearm whenever he/she wants. Furthermore, the pain is not caused instantly, that is, it grows slowly, and the process can stop before the pain becomes severe [5]. Moreover, the participant was free to quit this study at any time without any kind of prejudice. Thus, although pain is caused by this method, the findings can have a very high contribution to improving pain management and has no collateral physical or psychological effects. Nonetheless, there was a need to establish exclusion criteria [5].

It is of extreme importance to point out that all recommendations for data protection were followed, and that the integrity of the participants was guaranteed.

This study was approved by the Ethics and Deontological Council of the University of Aveiro (CED-UA-24-CED/2021).

#### 3.1.3. Experimental Procedure

Before starting the pain-inducing technique, the procedure was explained to the participants, and after removing the remaining doubts, an informed consent form was signed. Afterward, the individuals had to answer questions that addressed their personality attributes, health state, depression, and anxiety.

**Figure 1:**
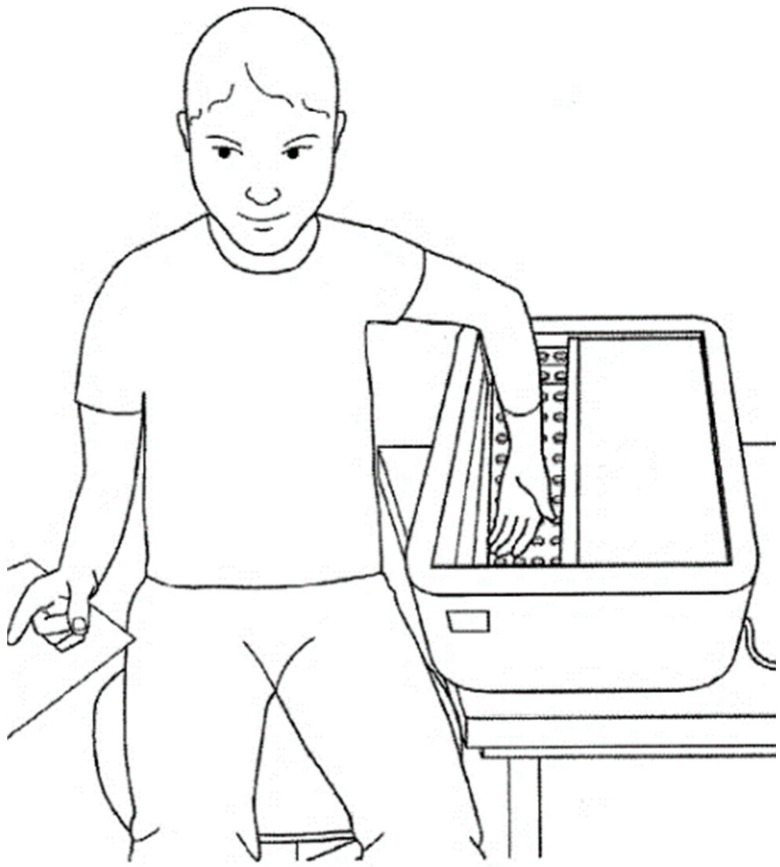
A participant has his left hand immersed in a tank containing circulating cold water.

During the whole procedure, the individual had to watch two kinds of videos that would provoke either fear or neutral emotions, which were classified by a research team of psychologists based on the emotion they elicit. In the first section of the protocol, a five-minute baseline was recorded, and while a film provoking a neutral feeling was shown, the participant had to be sat in a comfortable position, with their arm close to their body. Afterward, they were requested to put their nondominant hand and forearm in the warm water tank, as shown in Figure 1, for a period of two minutes, in order to assure that all the participants started the CPT with equal skin temperatures of 37 °C ± 1 °C.

In this part, the movie showed was fear-inducing or neutral-feeling-inducing, according to the first or second session, respectively. Approximately at the one minute and thirty seconds mark, their Blood Pulse (BP) was taken, and they were also asked to report their pain level using an NRS ranging from 0 to 10. After, the participants dipped their nondominant forearm in the cold water tank, with a temperature of approximately 7 °C ± 1 °C, and hence, the CPT commenced. Participants were asked to hold on as long as they could, with a time limit of two minutes. If they were not able to tolerate the pain, they were encouraged to inform the researchers of their desire to withdraw their forearm from the tank and, before doing so, to report their pain level. In the case that the participant was able to keep their hand in the tank for the complete duration, the maximum pain experienced was reported around the two minutes mark.

**Figure 2:**
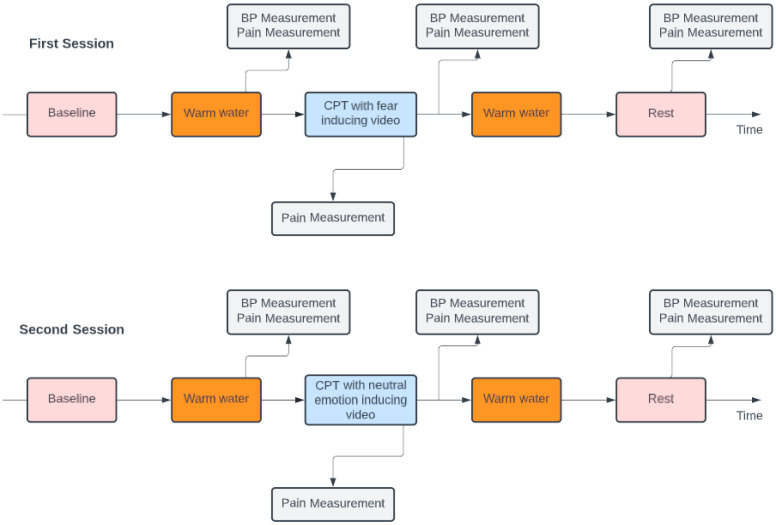
Scheme of the implemented protocol.

After the removal of the forearm from the cold water tank, the participant’s BP level was also assessed. The movie presented during the CPT depended on the session. In the first session, the video was fear inducing, and in the second session, the video was neutral feeling inducing. In the second session, the neutral videos were different from the first session, and the neutral videos during the CPT were also distinct from the video that played while the participants had their arm in the warm tank. Before the end of the protocol, the last segment took place where, at rest, the participant had to watch a five-minute neutral video while sitting in a comfortable position, without the forearm immersed. After two and a half minutes, their BP level was again monitored, and they were asked to report their pain level. The scheme of this experimental protocol can be observed in Figure 2.

#### 3.1.4. Data Collection Setup

The apparatus can be categorized into two sorts, the equipment to induce pain by exposure to cold water and the device to gather physiological data.

##### CPT Tank System

The apparatus utilized in studies that involve the CPT can be custom made, maintaining an approximately constant water temperature and a flow of water over the hand. In this case, the equipment was composed of two tanks, one had warm water and the other had cold water. Both tanks were isolated to avoid heat transfer between the warm or cold water and the environment. These tanks allowed us to have control over the variation in the temperature as there was a microcontroller to revert losses and keep the water tank’s temperature stabilized and a circulating water pump to avoid heating/cooling of the water and to guarantee that the water in touch with the participant was at the same temperature.

The purpose of the first specified tank was to ensure that every participant started the procedure under similar conditions, which prevented possible bias, while the second was used to induce pain in the participant.

This system was developed in the scope of an Integrated Master’s in Electronic and Telecommunications Engineering at the Department of Electronics, Telecommunications and Informatics (DETI), University of Aveiro.

##### Physiological Signal Collection

**Figure 3:**
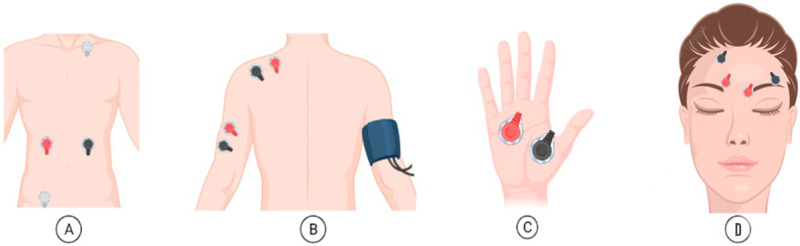
Placement of the electrodes to collect data: (**A**) ECG, (**B**) EMG in trapezius and triceps, (**C**) EDA, (**D**) EMG in corrugator and frontalis.

The device used to gather data was the Biosignalsplux Explorer tool kit, with a sampling frequency of 1000 Hz. This is a 4-channel toolbox that allows for wireless and high-quality acquisitions. This toolkit enables the simultaneous collection of data from 4 sensors, up to approximately 10 h of signal streaming at up to a 3000 Hz sampling rate and 16-bit resolution per channel. In this data protocol collection, two synchronized kits were used with seven sensors for EMG, ECG, EDA, and BP, and the signals were recorded at 1000 Hz. Figure 3 shows the placement of the sensors to record: (A) ECG, (B) EMG in the trapezius and triceps, (C) EDA, and (D) EMG in the corrugator and frontalis.

#### 3.1.5. Subjects

For this study, there was a total of 37 participants; in total, 14 were male, and the ages ranged from 19 to 25 years old (average age of 21.36 y.o. and standard deviation of 1.27 y.o). However, one of the individuals was removed owing to an equipment fault whereby it was not possible to perform the second session of the protocol, resulting in a total of 36 participants with valid data collected. The volunteers were recruited from the university student’s community and had to follow the inclusion criteria outlined in Section 2.1.

### 3.2. Methods for Data Analysis

After acquiring the data, the raw ECG had to be preprocessed for further analysis.

The data processing and analysis were performed using Spyder version 5.1.5, with SciPy version 1.6.0 [15], Neurokit2 version 0.2.1 [16], scikit-learn version 1.1.3 [17], and seaborn version 0.12.1 [18].

After acquiring the raw ECG, EMG, and EDA signals, they had to be preprocessed.

#### 3.2.1. ECG Filtering

The raw ECG signals were preprocessed before further analysis. There were several types of filters that could have been used to filter the signals, yet the infinite impulse response (IIR) was chosen due to its simplicity of construction and efficiency. Before filtering, the ECG signal was centered (i.e., the mean was subtracted from the signal).

The ECG was filtered to remove the artifacts caused by the baseline wander and high frequencies that did not contribute to the detection of the QRS complexes. The analysis of the ECG signals in the frequency domain showed that the frequency components of interest of most of the ECG signals were between 0.5 Hz and 40 Hz. Therefore, different orders of Butterworth bandpass filters, with these cutoff frequencies, were used to process the signals. Despite being an infinite impulse response filter, the Butterworth filter was chosen due to the insurance of a frequency response that is as flat as possible in the passband. Moreover, the nonlinear phase distortion was eliminated by processing all the entire ECG signals in both the forward and reverse directions. Thus, according to the mean absolute error and mean squared error that were computed from raw and filtered signals, the ECG signals were filtered with a band pass, cutting frequencies of 0.5 Hz and 40 Hz, a Butterworth filter of order 4, and the following transfer function:

$$H(z)=\frac{0.0131-0.0261z^{-2}+0.0131z^{-4}}{1-3.6504z^{-1}+5.0050z^{-2}-3.0586z^{-3}+0.7040z^{-4}}$$

**Figure 4:**
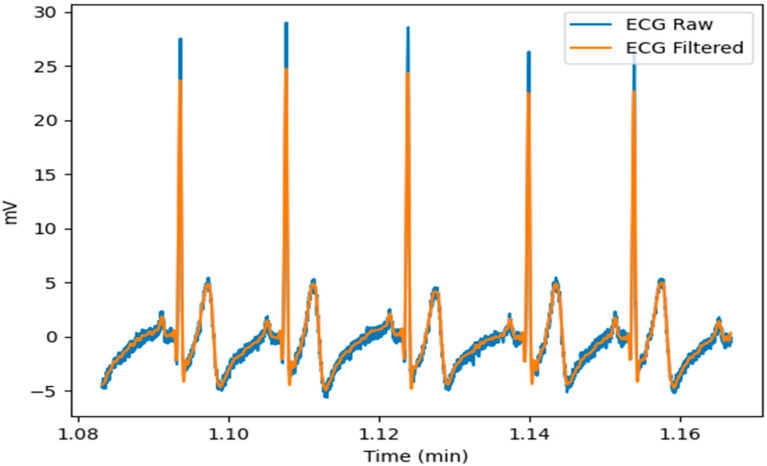
Overlay of filtered signal and original.

An example of the result of the filter is shown in Figure 4. As can be seen in this figure, there was a significant difference in noise between the ECG raw and the filtered ECG.

The data were then processed using the Neurokit2, a user-friendly program that provides easy access to complex biosignal processing methods [15]. The “ecg_process” method was used, returning the filtered signal; the completion of the auricular and ventricular phases; the positions of the peaks of the P, R, S, and T waves; as well as the onsets and offsets from the P, R, and T waves. Furthermore, this method can be used to calculate HRV features for time, frequency, and nonlinear domains.

Following signal processing, each signal was separated into five distinct epochs based on the triggers gathered during the experimental protocol, as illustrated in Figure 2. As a result, the signal was divided into a five-minute baseline recording, two minutes of the nondominant forearm in the warm water tank, the CPT, another two minutes of the nondominant forearm in the warm water tank, and the last five minutes of rest period.

#### 3.2.2. Feature Extraction and Normalization

**Figure 5:**
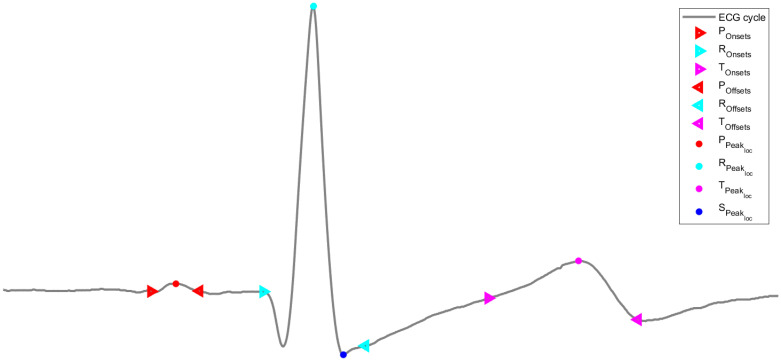
Location of the extracted peaks, onsets, and offsets of an ECG cycle.

The features used to train and test the machine learning models were extracted using sliding windows of 20 s periods with a 75% overlay. After identifying the positions of the peaks of the P, R, S, and T waves, as well as the onsets and offsets from the P, R, and T waves, shown in Figure 5, the following features were computed from the ECG signals:

- P_peaks_, R_peaks_, S_peaks_, T_peaks_: the number of peaks in the waves in each window;
- P_amplitude_, R_amplitude_, S_amplitude_, T_amplitude_: the average of the amplitude of the correspondent peak in the waves (which is given by the amplitude value of the ECG wave at the correspondent peak) in each window;
- P_distance_, R_distance_, S_distance_, T_distance_: the average of the distance (in samples) between consecutive peaks in each window (these distances are computed based on the sample of each peak);
- P_onsetamp_, R_onsetamp_, T_onsetamp_: the average of the amplitude of the correspondent peak onsets in the waves (which is given by the amplitude value of the ECG wave at the correspondent peak onset) in each window;
- P_offsetamp_, R_offsetamp_, T_offsetamp_: the average of the amplitude of the correspondent peak offsets in the waves (which is given by the amplitude value of the ECG wave at the correspondent peak offset) in each window;
- P_onoffdist_, R_onoffdist_, T_onoffdist_: the average of the distance (in samples) between consecutive peak onsets and peak offsets in each window (these distances are computed based on the sample of each consecutive peak onset and peak offset).

Prior to proceeding with the data analysis, the collected features were all normalized by dividing each epoch by the mean of the baseline.

#### 3.2.3. Classification Approaches

As the focus of interest relies on identifying the pain-inducing period from the baseline state, only ECG features from the baseline and the CPT epochs were used. Thus, the positive class (the class of interest) was considered to be the CPT epoch and the negative class corresponds to the baseline.

**Figure 6:**
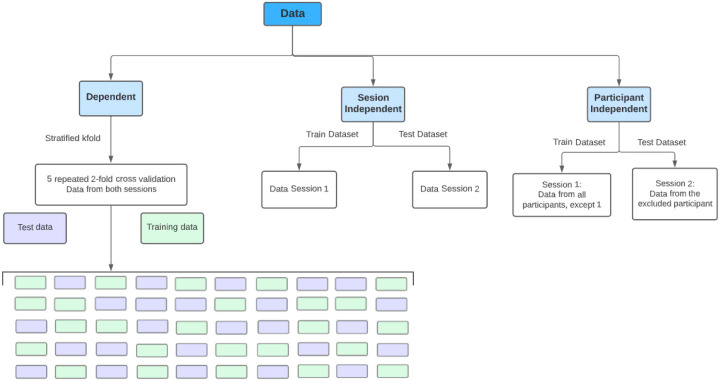
Scheme of the different approaches to divide data for model learning.

Furthermore, with regard to the training and testing strategy, the following approaches, illustrated in Figure 6, were considered:

1. Dependent approach: using all the data from both sessions, the models were trained and evaluated with 5 repetitions of a 2-fold cross-validation strategy. In this approach, data from the same participant in the same session can be shared between the training and test sets (although, the same data are not shared by both). In this approach, the training dataset had 2830 samples and the test dataset had 2831.
2. Session-independent approach: considering an analysis across both sessions, the training set consisted of data from all participants from the first session, whereas the test set consisted of data from all participants from the second session. Although in this approach, the data from different sessions are not shared between the training and test sets, and data from the same participants, in different sessions, are shared. Thus, this approach is not entirely participant independent, as the participants were the same in both sessions. In this approach, the training dataset had 2771 samples and the test dataset had 2890.
3. Participant-independent approach: In this approach, data from participants in the training set are not shared with the test set, not even from different sessions. Using the leave-one-out cross-validation (LOOCV) strategy, the training data was composed of the data from the first session of all participants except one, i.e., a model was built for each participant with their data from the second session serving as the test set. In this approach, the training dataset had, on average, around 2694 samples and the test dataset had, on average, approximately 80 samples.

#### 3.2.4. Machine Learning Algorithms

In order to classify pain, eight distinct machine learning algorithms were used to analyze the data from various perspectives, namely AdaBoost, DT, kNN, LDA, Logistic Regression (LR), RF, and SVM (with a linear kernel and a Radial Basis Function kernel), using the default parameters as shown in Table 1.

A DT is a machine learning algorithm for classification and regression, where the data are used to deduce if–then–else rules. These rules grow more complicated as the depth of the tree increases. Thus, a classification DT structure can be visualized as an inverted tree, starting at the root, with internal nodes and branches, and ending in leaves with a target class. The advantages of DT include their straightforward understanding of rules, lack of data normalization requirements, lower processing cost, and handling of numerical as well as categorical data [19,20].

An RF is an ensemble of randomized DT, each of them grown using a bootstrap sample of the original data and using a randomized subset of features to choose the best split at each node. Thus, the forest is composed of several different DT, and, in a classification problem, the outcome consists of a majority vote [19,20].

AdaBoost, also an ensemble of models, is a strategy for repeatedly applying new data to weak estimators. This includes raising weights for incorrectly predicted training observations and reducing weights for correctly predicted training observations. As a result, with each successive iteration, to raise the performance, the estimator focuses on training observations that had incorrect predictions in the prior iteration [20].

The kNN approach is based on computing distances from neighbors, and the main principle is to locate a specified k number of training observations that are closest to the new observation and then use these nearest neighbors to determine the target for this new observation [19,20].

Another model that was trained was LDA, which tries to maximize class separability by learning linear decision boundaries. It is computationally efficient with no need for hyperparameter adjustments [21]. Likewise, with low computational complexity, LR is also a linear model for classification with the ability to provide probabilities describing the possible outcomes and is less sensitive to outliers [19,20].

SVM is an algorithm that tries to optimize a margin between two classes. SVM’s key merits include its efficacy on high-dimensional data and datasets with more features than observations, as well as its low memory consumption due to the usage of support vectors. When the data are not linearly separable, they are mapped into a higher dimensional space before the optimization of the decision boundary, and a broad range of kernel functions can be used to achieve this transformation [19,20].

#### 3.2.5. Evaluation Metrics

Six evaluation metrics were used to assess the performance of each model: the accuracy score, the balanced accuracy score, the F1 score, the Matthews correlation coefficient, the precision score, and, lastly, the recall score. The accuracy score calculates the percentage of right predictions out of the total number of guesses. When the data are imbalanced, however, the accuracy might be misled, and the balanced accuracy score allows this problem to be avoided by computing the average of recall and specificity. The precision score is the percentage of correct positive predictions out of all positive forecasts. The recall score indicates how many positive predictions were retrieved. The F1 score is a balance between the two most recent metrics, and it allows for the avoidance of an erroneous prediction accuracy and works better with imbalanced classification data. Finally, the Matthews correlation coefficient (MCC) considers all four values in the confusion matrix, and a high value indicates that both classes are well predicted, even if one class is disproportionately under- or overrepresented.

## 4. Results

The purpose of this study was to analyze the physiological changes conveyed by the ECG caused by pain induction in autonomic reactivity with different emotion elicitation. The hypothesis is that the different emotional contexts should alter the pain perception of the participants, while the physiological response remains the same.

The majority of the participants participated in the CPT for more than one minute, with only ten participants not holding for the entire time. The participants in the first session held their arm in the tank for a mean time of one minute and fifty-three seconds, and the participants in the second session held it for a mean time of one minute and forty seconds.

### 4.1. Perception of Pain under Different Emotional Contexts

**Figure 7:**
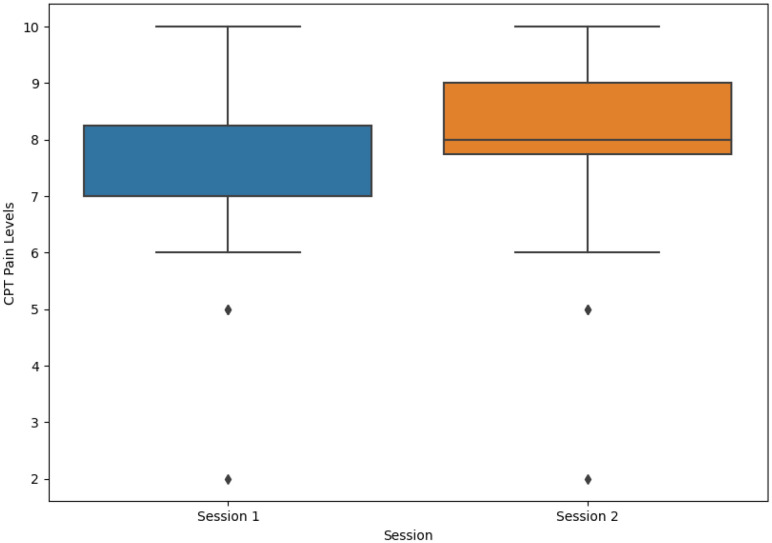
Pain levels reported by participants before the end of the CPT for both sessions.

As previously stated, participants were required to report their level of pain at three different stages. Prior to being subjected to the CPT, none of the participants reported pain during the initial evaluation. The level of pain was assessed again before the end of the CPT as the subjects were required to report their current pain. In the first session, the average value reported was 7.39 ± 1.64 (mean ± standard deviation (SD)) and the median was seven, and in the second session, the average value reported was 7.80 ± 1.60 (mean ± SD) and the median was eight. For both sessions, the average value reported was 7.58 ± 1.63 (mean ± SD) and the median was eight. Most participants felt an immediate improvement in their pain levels after removing their arm from the cold water. Participants reported their current level of pain on the last pain assessment, and the values dropped significantly. The distribution of pain levels reported by patients before the end of the CPT is depicted in Figure 7.

The implemented protocol varied depending on the CPT session: the video shown during the CPT in the first session was designed to elicit fear, whereas the video shown in the second session was designed to elicit a neutral emotion. According to the reported pain level before the end of the CPT and comparing the boxplots in Figure 7, the difference seemed to be minimal. A paired t-test with pain levels reported from both sessions returned a *p*-value of 0.280, revealing that there was no indication of statistical difference between both sessions.

### 4.2. Physiological Response of Pain under Different Emotional Contexts

Taking into consideration the three approaches proposed, different classifiers were trained with the different data organization methods with the purpose of analyzing the participant and session dependency influence in the classification results.

#### 4.2.1. Dependent Approach

Table 2 shows the performance metrics of the models trained with this approach.

According to the performance of each model, the results may be divided into three groups. To begin, the model with the worst results was LR, which had an accuracy of 87% and a recall score of 45%. This indicates that 55% of the CPT samples remained to be predicted; however, a total of 94% of the CPT predictions made by this model were correct, suggesting a high capability in classifying the negative samples (from the baseline) but a poor ability to classify the class of interest, which was fewer samples. The MCC was the lowest, with a value of 0.59, indicating that the predictions had a slight correlation with the true class.

Second, the models that presented an average overall performance were the LDA, SVM-lin, and SVM-rbf, which had an accuracy of 87% to 90%. Aside from that, these models correctly predicted slightly more than half of the CPT data, ranging from 51% to 65%. Furthermore, the MCC ranged between 0.61 and 0.72, which was greater than the LR, indicating a correlation between the anticipated and genuine data.

Finally, AdaBoost, DT, kNN, and RF yielded the best outcomes. The accuracies of these models ranged between 87% and 98%, with kNN having the lowest value and the RF having the highest. These models predicted almost all of the CPT data, between 81% and 95%, whereas the models were correct between 94% and 98% of the time. Additionally, the MCC ranged between 0.91 and 0.97, suggesting that these were the techniques with the strongest correlation between the real and expected data.

**Figure 8:**
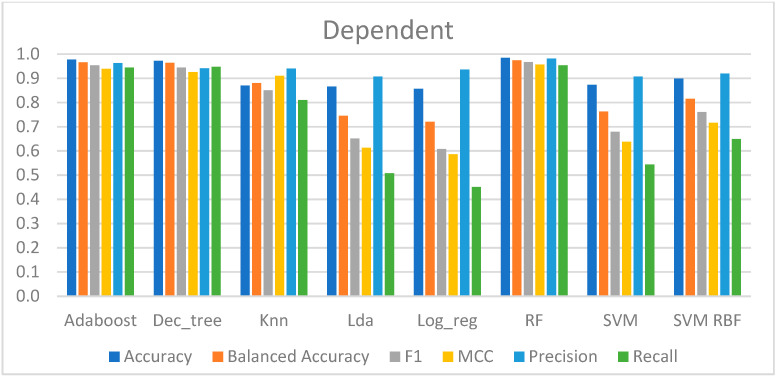
Metrics for performance evaluation of the dependent approach.

Ultimately, these models, trained and evaluated with five repetitions of a stratified two-fold cross-validation strategy, were able to recognize pain for the binary classification between the baseline and the CPT, as can be observed in Figure 8.

#### 4.2.2. Session-Independent Approach

Table 3 provides the performance metrics for the models trained using this approach.

Taking an overview, the models that were best suited to the data were AdaBoost, RF, DT, and SVM-rbf, with an accuracy ranging from 91% to 97%. The recall score of these four approaches was quite high, and in AdaBoost, RF, and SVM-rbf, it was very near to one, indicating that they could predict nearly all of the CPT data. The precision of these approaches was not as great as the recall, with values ranging from 79% to 90%, which means that these models correctly predicted at least 79% of all pain (positive) predictions. To summarize these methodologies, the MCC produced revealed a very high correlation between the real class and the predicted data, with the lowest value being 0.784 and the greatest being 0.928.

In terms of performance, the other approaches were relatively comparable but not as good as the ones stated above. Their overall performance was acceptable, with an accuracy range of 72% to 84.4%. However, these results mask the true performance since the recall score was only between 52% and 68%, implying that about half of the CPT data were incorrectly predicted. However, their precision in predicting the CPT data was higher, ranging between 67% and 80%. Aside from that, the predicted data showed only a little connection with the real class, as the MCC was between 0.566 and 0.61.

**Figure 9:**
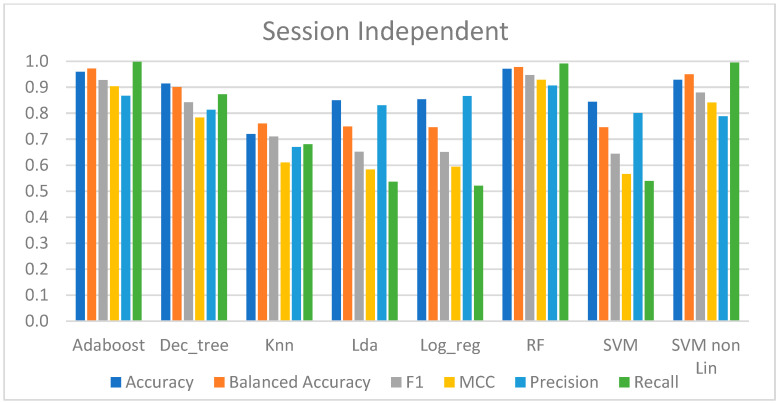
Metrics for performance evaluation of session-independent approach.

To summarize, the highest performing models based on this session-independent approach were able to discriminate between the baseline and pain, as shown in Figure 9.

#### 4.2.3. Participant-Independent Approach

In this approach, one model per participant was trained with data from the first session from the remaining participants, and the performance was evaluated with data from the participant in the second session. Therefore, the average performance of all participants is shown in Table 4.

The LDA, kNN, LR, SVM-lin, and SVM-rbf models produced the worst results. Although these models had over 80% accuracy, except kNN with 63%, and precision scores ranged from 74% to 83%, implying that at least 74% of all the CPT predictions were correct, and the recall score only ranged from 39% to 65%, implying that in almost all these methods, at least half of the CPT data were incorrectly predicted. However, there was a slight correlation between the predicted data and the class of interest, since the MCC ranged between 0.47 and 0.7. From these models, the SVM-rbf and kNN presented the best performance metrics.

On the other hand, AdaBoost, DT, and RF had high performances in classifying pain, with an accuracy of over 93%, and predicted at least 87% of the class of interest. Furthermore, 87% to 97% of all the predictions were correct, and these had a high correlation with the true classes, as the MCC was at least 0.86.

**Figure 10:**
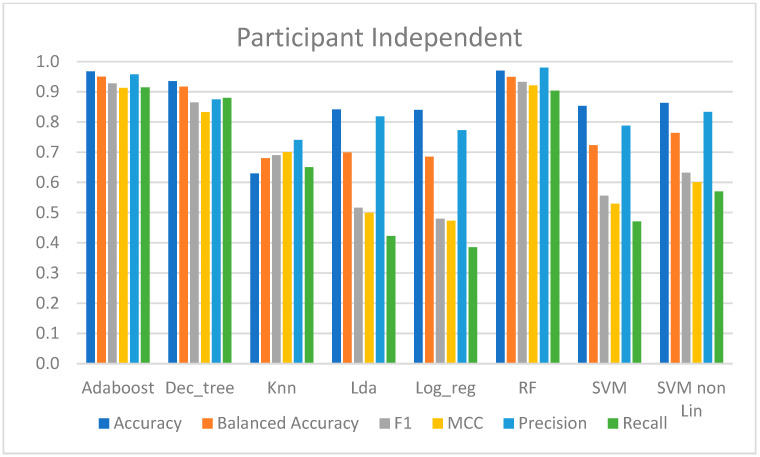
Metrics for performance evaluation of participant-independent approach.

Figure 10 presents the computed metrics for evaluating the performance of the models trained with this approach.

#### 4.2.4. Feature Importance

Depending on the model, the importance of each feature was determined with different methods. LDA and LR are models that employ a set of coefficients in the weighted sum to generate a prediction, and these coefficients can be used as their feature relevance score. The importance scores provided by RF, Adaboost, and DT are based on a decrease in the criterion used to choose the best split at each node, which in this context is gini. Finally, the relevance of features for SVM and kNN was determined by feature evaluation based on the permutation of features (using the scikit learn function permutation_importance).

Thus, for each model, it was possible to deliver a list with the score assigned to each feature depending on how effective they were in predicting each sample. However, due to the approach to derive the scores of the features, the lists were not comparable across all models. As a result, the values were normalized for all of them to be between 0 and 1.

$${feature}_{scaled}=\frac{feature-{feature}_{min}}{{feature}_{max}-{feature}_{min}}$$

##### Dependent Approach

**Figure 11:**
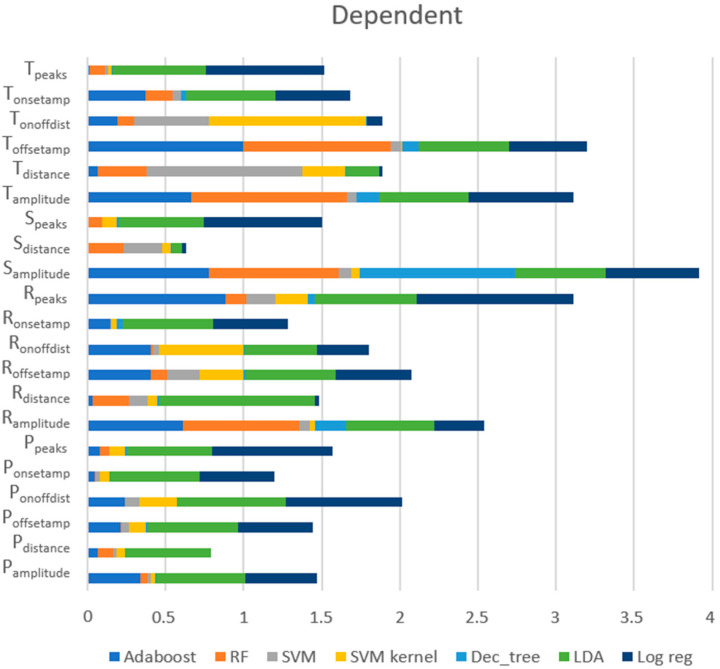
Importance of features for each model using the dependent approach.

Figure 11 exposes the scores of each feature provided by each model, showing that the most relevant features for this approach were (1) S_amplitude_ (the amplitude of the S peaks), (2) T_offsetamp_ (the amplitude of the T_offset_), (3) R_peaks_ (the number of the R peaks), (4) T_amplitude_ (the amplitude of the T peaks), and (5) R_amplitude_ (the amplitude of the R peaks). These five features were the ones with the best scores for RF and AdaBoost, which were models with very good performances. However, regarding the top five features, LDA gave more importance to other features, such as the distance between the R peaks (R_distance_) and the distance between the consecutive onset and offset of the P wave (P_onoffdist_), which may have been the cause for poor performance.

##### Session-Independent Approach

**Figure 12:**
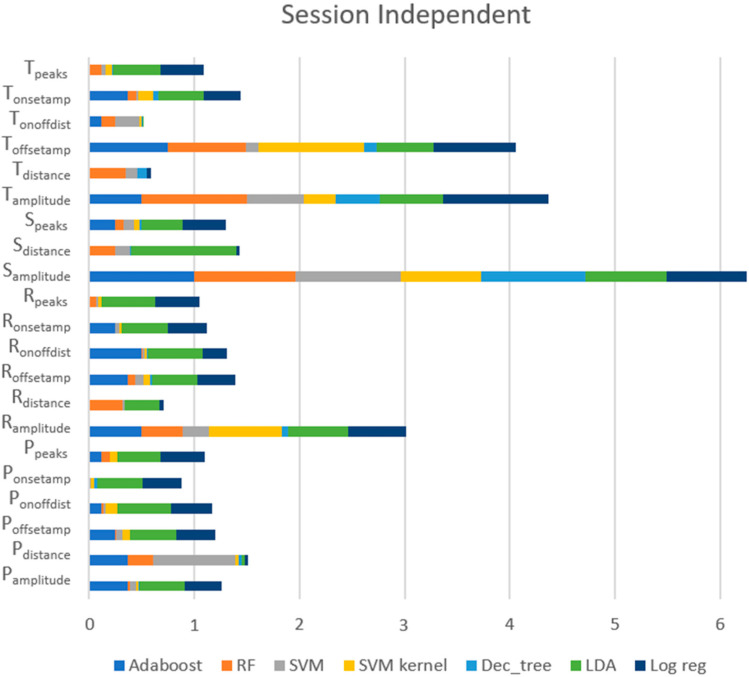
Importance of features for each model using the session-independent approach.

Figure 12 shows the ratings of each feature provided by each model, exposing that the most valued features for this approach, across all models, were (1) S_amplitude_ (the amplitude of the S peaks), (2) T_amplitude_ (the amplitude of the T peaks), (3) T_offsetamp_ (the amplitude of the T_offset_), (4) R_amplitude_ (the amplitude of the R peaks), and (5) P_distance_ (the distance between the consecutive P peaks). For RF and AdaBoost, which were models with excellent performance, these five features remained the best. However, for the top five features, LR and LDA, which had poor outcomes, scored higher than for other features, such as R_distance_ and S_distance_.

##### Participant-Independent Approach

**Figure 13:**
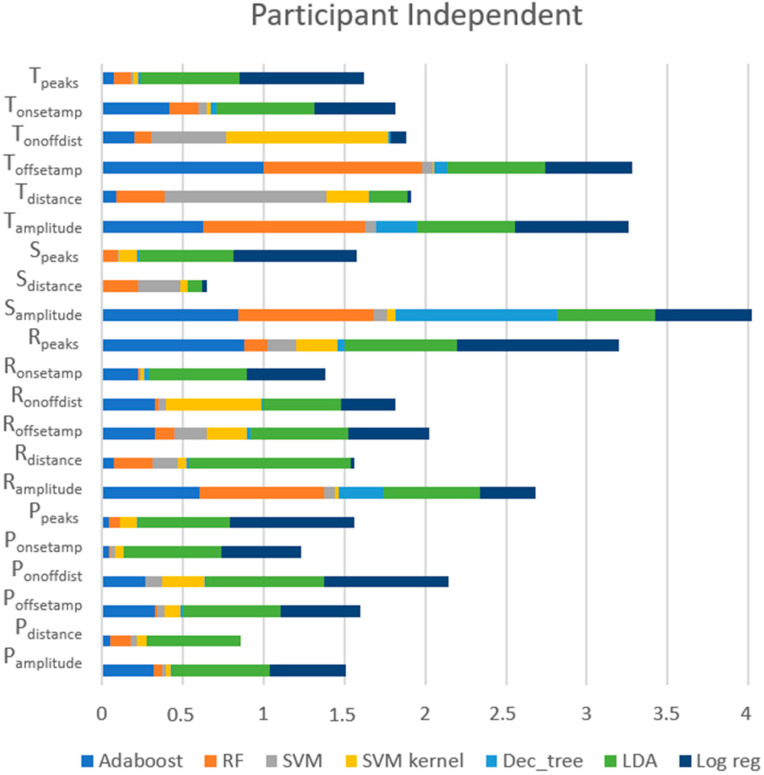
Importance of features for each model using the participant-independent approach.

Figure 13 presents the scores of each feature provided by each model in this approach, revealing that the most important features, across all models, were (1) S_amplitude_ (the amplitude of the S peaks), (2) T_offsetamp_ (the amplitude of the T_offset_), (3) T_amplitude_ (the amplitude of the T peaks), (4) R_peaks_ (the number of R peaks), and (5) R_amplitude_ (the amplitude of the R peaks). For RF and AdaBoost, which were models with high performances, these five features continued to have the highest scores. However, the top five features for LR and LDA, which had poor outcomes, also included R_distance_ and S_distance_.

In general, the five most valued features were relatively similar across all approaches. The feature with the most relevance for all methods was S_amplitude_, whereas T_amplitude_, T_offsetamp_, and R_amplitude_ remained in the top five.

## 5. Discussion

Concerning the role of emotional conditions on pain perception, the average level of pain in the first session was lower than in the second session: in the first session, it was 7.39, and in the second session, it was 7.80 (on a scale from 0 to 10), as shown in Figure 7. These similar results, with only a 0.41 difference and with the statistical test revealing no differences in the pain score between both sessions, support the notion that pain perception was not influenced by the different emotional contexts. These results may indicate that emotions were not adequately elicited, which could be due to two factors: the participant was focused on the pain felt and thus did not pay enough attention to the video, or the participant already knew the video and thus it was unable to elicit fear. Indeed, several participants indicated that they were unable to view the emotion elicitation video during the CPT as they were more focused on dealing with the pain than on being attentive, and some also reported knowing the video shown. Based on these statements and the equality of pain perception, it was assumed that emotion elicitation was not achieved.

With respect to the physiology of pain under different emotional contexts, with the dependent approach, a balanced accuracy of up to 97.4% was obtained, and with the session-independent approach, a balanced accuracy of up to 97.7% was reached. These results support that the physiological response conveyed by the several analyzed ECG features remained similar across different emotional contexts.

Moreover, considering the session-independent approach outlined in Section 4.2.2 and looking at the high performance of each model in Table 3 and Figure 9, it is possible to conclude that, despite the fact that not all models performed well, AdaBoost and RF presented a high performance in classifying pain. This was evidenced by a recall score close to one, indicating that these models correctly predicted almost all the CPT samples, and a precision score between 87% and 90%, indicating that only about 10% of the CPT predictions made were incorrect.

Considering the participant-independent approach, the findings of which are provided in Section 4.2.3, it was feasible to verify that pain could be appropriately classified regardless of the availability of the participant’s information. As previously indicated, data from a participant in the second session were used to assess the performance of the corresponding model, trained with data from the first session of all the participants except the participant being tested. As a result, as shown in Table 4 and Figure 10, some models performed far better than others. These were the AdaBoost and RF, which did not correctly predict about 10% of the CPT data and were correct in 95.7% to 98% of the predicted CPT data. These findings support the notion that there is no requirement for participant input during the training phase, and hence these trained models might be utilized to categorize the existence of pain in subjects who are not present in the training phase.

**Figure 14:**
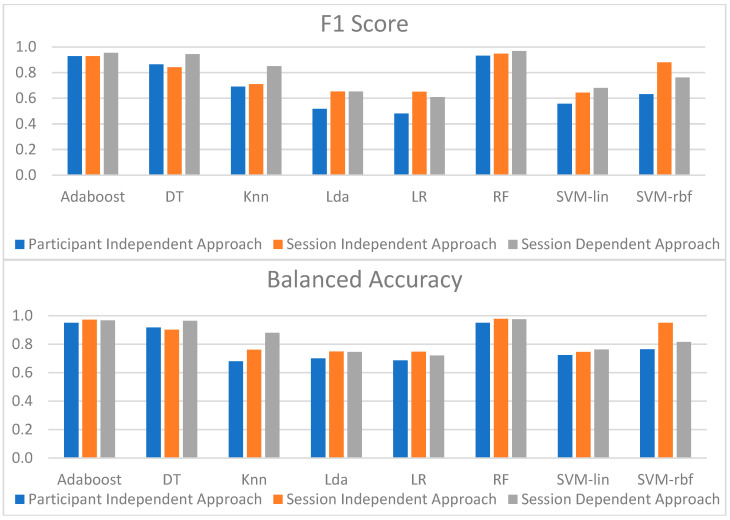
Obtained F1 (**top**) and balanced accuracy (**bottom**) from all models using the three different approaches.

Figure 14 shows the overall performance of the models for the three approaches. Since F1 reflects both recall and precision scores, and balanced accuracy is more suitable for unbalanced datasets than accuracy, these metrics were chosen to compare the models’ performance.

When the performance of each model across the three approaches was evaluated, it was possible to conclude that the dependent strategy gave the best results. However, this slightly increased performance may have been due to the cross-validation strategy. Accordingly, the model that provided the best results was RF, suggesting that it could reliably identify almost all the CPT data across the different approaches.

The findings achieved with the different models using the three approaches were all fairly comparable. The model with the best results for the session-independent method was also RF, with a recall score of 99.1% and a precision of 90.6%. Furthermore, the model with the highest results for the participant-independent method was RF, with a recall score of 90.4% and a precision of 98%. Although the RF model was the best for the three approaches, AdaBoost’s metrics were also extremely excellent, and in some situations, they were even higher than those of RF. As a result, in all three approaches, RF and AdaBoost were the models that could best classify pain, which may be supported by the importance that both give to the top five features among all the extracted features.

With regards to the execution time, it must be stressed that although the LDA and LR models had the worst performance results for pain recognition for the three machine learning approaches, they had lower execution times: 0.34 s, 0.06 s, and 0.83 s; and 0.78 s, 0.09 s, and 2.78 s, respectively. DT, AdaBoost, and RF presented intermediate runtimes: 0.79 s, 0.09 s, and 2.25 s; 5.74 s, 0.62 s, and 15.79 s; and 11.23 s, 1.28 s, and 32.15 s, respectively, whereas SVM and kNN presented higher execution times with the three approaches.

## 6. Conclusions and Further Work

This work showed the implementation of a protocol for data collection, with the primary goal of creating a database with different physiological signals, frontal video, and self-report questionnaires of participants who were subjected to two sessions of a pain-inducing task while different emotional states were being elicited. It was feasible to establish a modest database, with 36 participants but with a lot of varied data, that allowed for the analysis of the influence of pain in the human body, as well as the impact emotions have on the perception of pain felt using the aforementioned procedure. However, it was likely not viable to identify the emotional effect, or even the emotion itself, because, according to several participant reports, they were unable to pay close attention to the exhibited movies during the CPT. Additionally, the analysis of the pain scores acquired during the CPT in both sessions supported the idea that the elicitation of emotions was not achieved successfully, as the statistical test on the pain perception between sessions did not reveal significant differences. This leads to the need for further reasoning for better emotional elicitation while inducing pain.

Despite failing in the emotion elicitation, it was feasible to build machine learning models that are able to recognize pain based on physiological information from the ECG, regardless of the cultural variations, given the participants came from various cities around the country. Moreover, in this regard, it is also possible to derive interesting implications from the examination of each evaluation strategy. It was possible to identify and separate the CPT data from the baseline using the dependent approach, in which the data were split using stratified cross-validation, with five repetitions of two folds, indicating that the trained models were successful at detecting pain. This approach served the purpose of comparison with the independent approaches.

The results from the session-independent approach allowed us to confirm that there seemed to be no difference in the physiological response of ECG between the sessions because when data from the first session were used to train the models and the data from the second session were utilized for testing, a similar performance was achieved by the top models (RF, DT, and AdaBoost). Finally, the results with the participant-independent method showed that there was no need for the participants’ information since the models correctly identified pain.

Several topics can be tested and improved in future development. As previously stated, the emotion elicitation was unsuccessful since participants indicated that they were unable to pay attention to the fear-inducing videos as they were focused on the pain they were experiencing. As a result, the protocol’s design should be altered to isolate emotion elicitation from the pain-inducing activity, for example, the video inciting fear should be played prior to the CPT.

Longer CPT signals are also necessary to analyze HRV measures that require longer durations to be meaningful, as well as contribute to a less unbalanced dataset. Given this, a larger number of participants is also advisable.

The collected database can also be used to perform a more complete study, such as the study of psychophysiological correlates on pain, leading to a better and more comprehensive understanding of pain mechanisms. Moreover, this database also supports multimodal approaches, taking into account the physiological response of the other collected signals, allowing one to analyze and compare the performance with the recognition of pain based solely on information conveyed by the ECG. Other data mining approaches, such as feature selection and feature normalization, can also be used before providing input to each model. In this study, feature normalization was applied to the data, and different approaches could be employed to allow for a comparison between the performance of the trained models.

Finally, hyperparameter optimization and the development of individual models adapted to each participant should also be taken into consideration to enhance the recognition of pain, and it may provide a step further to personalized medicine.

## Figures and Tables

**Table 1:** Default parameters, in scikit-learn’s implementation, of the machine learning algorithms used.

	Default Parameters (in Scikit-Learn’s Implementation)
**AdaBoost**	base estimator: DecisionTreeClassifier, n_estimators: 50, learning_rate: 1.0, algorithm: real boosting algorithm.
**DT**	criterion: gini, splitter: best, max_depth: None (DT is expanded until all leaves are pure or until all leaves contain less than min_samples_split samples), min_samples_split: 2, min_samples_leaf: 1, min_weight_fraction_leaf: 0.0, max_features: None (consider all features when searching the best split).
**kNN**	n_neighbors: 5, radius: 1.0, algorithm: ‘auto’ (attempt to decide the most appropriate algorithm based on the values passed to fit method), metric: ‘minkowski’, p: 2 (using the Euclidean distance as metric).
**LDA**	solver: ‘svd’, shrinkage: None, priors: None, n_components: None (minimum between NumberOfClasses-1 and NumberOf Features), tol: 1.0 × 10^−4^ (absolute threshold for a singular value of the data matrix to be considered significant).
**LR**	penalty: ‘l2’, dual: False (uses the primal formulation), tol: 1 × 10^−4^ (tolerance for stopping criteria), C: 1.0 (inverse of regularization strength), fit_intercept: True (adds a constant to the decision function), intercept_scaling: 1, class_weight: None (all classes have weight one), solver: ‘lbfgs’, max_iter: 100 (maximum number of iterations taken for the solvers to converge), multi_class: ‘auto’, warm_start: False.
**RF**	n_estimators: 100, criterion: ‘gini’, max_depth: None, min_samples_split: 2, min_samples_leaf: 1, min_weight_fraction_leaf: 0.0 (the minimum weighted fraction of the sum total of weights required to be at a leaf node), max_features: ‘sqrt’ (the number of features to consider when looking for the best split), max_leaf_nodes: None (unlimited number of leaf nodes), min_impurity_decrease: 0.0, bootstrap: True, oob_score: False (do not use the out-of-bag samples to estimate the generalization score), warm_start: False, class_weight: None, ccp_alpha: 0.0, max_samples: None (considers bootstrap samples of the size of the data matrix).
**SVM-lin** and **SVM-rbf**	C: 1.0 (regularization parameter, the strength of the regularization is inversely proportional to C), kernel: ‘linear’ and ‘rbf’ (for SVM-lin and SVM-rbg, respectively), gamma: ‘scale’, shrinking: True, probability: False (disables probability estimates), tol: 1 × 10^−3^ (tolerance for stopping criterion), cache_size: 200, class_weight: None, max_iter: −1 (no limit on iterations within solver), decision_function_shape: ‘ovr’, break_ties: False (the first class among the tied classes is returned).

**Table 2:** Metrics for performance evaluation of the dependent approach (for each metric, the best performance results are identified in bold).

	AdaBoost	DT	kNN	LDA	LR	RF	SVM-lin	SVM-rbf
**ACCURACY**	0.977	0.972	0.870	0.865	0.856	**0.984**	0.873	0.899
**BALANCED** **ACCURACY**	0.966	0.964	0.880	0.745	0.720	**0.974**	0.762	0.815
**F1**	0.954	0.944	0.850	0.651	0.608	**0.967**	0.679	0.761
**MCC**	0.939	0.925	0.910	0.613	0.586	**0.957**	0.637	0.717
**PRECISION**	0.963	0.941	0.940	0.907	0.936	**0.981**	0.907	0.920
**RECALL**	0.944	0.947	0.810	0.508	0.451	**0.953**	0.543	0.649

**Table 3:** Metrics for performance evaluation of the session-independent approach (for each metric, the best performance results are identified in bold).

	AdaBoost	DT	kNN	LDA	LR	RF	SVM-lin	SVM-rbf
**ACCURACY**	0.959	0.914	0.720	0.849	0.853	**0.971**	0.844	0.928
**BALANCED** **ACCURACY**	0.971	0.900	0.760	0.749	0.746	**0.977**	0.746	0.950
**F1**	0.928	0.842	0.710	0.652	0.650	**0.947**	0.644	0.879
**MCC**	0.904	0.784	0.610	0.583	0.594	**0.928**	0.566	0.841
**PRECISION**	0.867	0.813	0.670	0.831	0.866	**0.906**	0.800	0.788
**RECALL**	**0.997**	0.872	0.680	0.536	0.520	0.991	0.539	0.995

**Table 4:** Metrics for performance evaluation of the session-independent approach (for each metric, the best performance results are identified in bold).

	AdaBoost	DT	kNN	LDA	LR	RF	SVM-lin	SVM-rbf
**ACCURACY**	0.967	0.935	0.630	0.841	0.839	**0.970**	0.853	0.863
**BALANCED** **ACCURACY**	**0.950**	0.917	0.680	0.699	0.685	0.949	0.723	0.763
**F1**	0.928	0.864	0.690	0.516	0.480	**0.932**	0.556	0.632
**MCC**	0.913	0.832	0.700	0.500	0.473	**0.921**	0.530	0.600
**PRECISION**	0.957	0.874	0.740	0.818	0.773	**0.980**	0.787	0.833
**RECALL**	**0.914**	0.879	0.650	0.422	0.385	0.904	0.471	0.570

## Data Availability

The data are protected by the GDPR and cannot be publicly available.
